# Molecular epidemiology and collaboration of siderophore-based iron acquisition with surface adhesion in hypervirulent *Pseudomonas aeruginosa* isolates from wound infections

**DOI:** 10.1038/s41598-022-11984-1

**Published:** 2022-05-12

**Authors:** Hamed Tahmasebi, Sanaz Dehbashi, Mona Nasaj, Mohammad Reza Arabestani

**Affiliations:** 1 School of Medicine, Shahroud University of Medical Sciences, Shahroud, Iran; 2 Department of Laboratory Sciences, Varastegan Institute of Medical Sciences, Mashhad, Iran; 3 Microbiology Department, Faculty of Medicine, Hamadan University of Medical Sciences, Pajoohesh Junction, Hamadan, Iran; 4 Nutrition Health Research Center, Hamadan University of Medical Sciences, Hamadan, Iran

**Keywords:** Bacteriology, Biofilms, Microbiology

## Abstract

Iron/siderophore uptake may play an important role in the biofilm formation and secretion of extracellular proteins in *Pseudomonas aeruginosa* isolates. In the present study, the role of siderophores, heme, and iron regulatory genes in the virulence of *Pseudomonas aeruginosa* isolates collected from wound infection was investigated. Three hundred eighty-four (384) swab samples were collected from wound infection and identified by phenotypic methods. The quantitative real-time PCR (qRT-PCR) method was evaluated for the gene expressions study. Multi-locus sequence typing (MLST) was used to screen unique sequence types (ST) and clonal complexes (CC). Fifty-five (55) *P. aeruginosa* isolates were detected in all swab samples. Also, 38 (69.1%) isolates formed biofilm. The prevalence of virulence factor genes was as follows: *plcN* (67.2%), *exoY* (70.9%), *exoA* (60.0%), *phzM* (58.1%), *plcH* (50.9%), *lasB* (36.3%), *aprA* (69.1%), *lasA* (34.5%), *nanI* (74.5%), *exoU* (70.9%), *exoS* (60.0%), *exoT* (63.6%) and *algD* (65.4%). According to qRT-PCR, genes regulating iron uptake were highly expressed in the toxigenic isolate. The highest expressions levels were observed for *hemO, hasR,* and *pvdA* genes in the biofilm-forming isolates. The MLST data confirmed a high prevalence of ST1, ST111, and ST235, with six, five, and 12 clusters, respectively. ST235 and ST1 were the most present among the biofilm-forming and toxigenic strains. Also, the *nuoD* gene with 54 and *guaA* with 19 showed the highest and lowest number of unique alleles. We demonstrated that iron/siderophore uptake is sufficient for biofilm formation and an increase in the pathogenesis of *P. aeruginosa*. These results suggest that the iron/siderophore uptake system may alter the MLST types of *P. aeruginosa* and predispose to bacterial pathogenesis in wound infections.

## Introduction

*P. aeruginosa* is known as the causative agent of an extraordinary spectrum of diseases in humans. The pathogenesis of wound infection of *P. aeruginosa* is attributed to its ability to produce several virulence factors (VFs)^1,2^. Exotoxins are essential VFs and are highly toxic to host cells. Exotoxins A, Y, S, and U are encoded by *exoA*, *exoY*, *exoS*, and *exoU* genes, respectively^2,3^. Also, exotoxin T causes delays in the healing process in wound infections, potentially leading to invasion and dissemination into the bloodstream^4^.

Pyoverdine is produced by *P. aeruginosa* and scavenges iron from the bacterial extracellular environment, and transfers it back into the cell. First identified the siderophore role of pyoverdines reported by Meyer and Abdallah^5^. Pyoverdine production is positively controlled by *pvdS* and *pvdA* genes, whose expression increases in response to iron scarcity. Also, pvdS is a sigma extracytoplasmic function, which is probably associated with the putative anti-sigma factor FpvR^6^. The *pvdS* modulates the expression of endopeptidase (*prpL*), and exotoxin A (*toxA*)^7,8^. Pyoverdine has a high affinity for iron and is delivered into bacterial cells through a tonB-dependent receptor (TBDR). TBDRs are essential in the acquisition of ferric substances and their transport into the cell cytoplasm^8^. TBDRs are directly responsible for ferrisiderophore transport in the cytoplasm. TBDRs only transport siderophores in the periplasm, the transport through the inner membrane involves other proteins^9,10^. The *pchABCD* are involved in pyochelin biosynthesis and the cytoplasmic transcriptional regulator *pchR*^11^. However, the *fptA* gene is the essential Fe (3+)-pyochelin receptor^12^. Heme proteins in the host are also targeted directly by *P. aeruginosa*; this occurs via two systems known as Has and Phu^13^.

Under iron-limiting conditions, the expression of quorum sensing (QS) systems is elevated^11,14^. Also, the production of pyocyanin is regulated directly by the Las and QS systems. Biofilm formation involves many different group activities, such as QS and iron siderophore production^15^. These activities can determine the structure and antibiotic resistance of biofilms. However, another intriguing phenomenon that further demonstrates this interdependent relationship between biofilm formation and iron acquisition is the ability of biofilms to store iron^16^.

Furthermore, *P. aeruginosa* may be able to survive in this environment due to the presence of some unique virulence factors, but the microorganism's iron acquisition in the new environment is likely to be suboptimal^8,13^. Iron acquisition requires a process of adaptation within this environment, including changes in pre-existing genes^8^. The mutations are commonly found in genes associated with virulence, antibiotic resistance, iron acquisition, and global regulator genes These genes have a role in the host adaptation in wound infection. They are essential for regulatory networks and central metabolism, acquisition of antibiotic resistance, and loss of extracellular VFs^8,13^. As a consequence, pathoadaptive mutations are projected to enhance bacterial virulence in the new environment, increasing the microbial population's growth and antibiotic resistance rates, and therefore driving the evolution of a relatively benign microorganism towards a more pathogenic lifestyle^17,18^.

However, the role and relationship of iron/siderophore acquisition systems in the pathogenesis, sequence types (ST), and clonal complex (CC) of *P. aeruginosa* in wound infection are unclear. Therefore, in this study, we investigate the role and effect of iron acquisition systems in the pathogenicity of *P. aeruginosa* in wound infection isolates.

## Results

In 384 samples of wound swabs, 55 (14.3%) isolates of *P. aeruginosa* were collected, including 38 isolates from females (69.1%) and 19 isolates (34.5%) from males.

### Antibiotics susceptibility patterns

**Figure 1:**
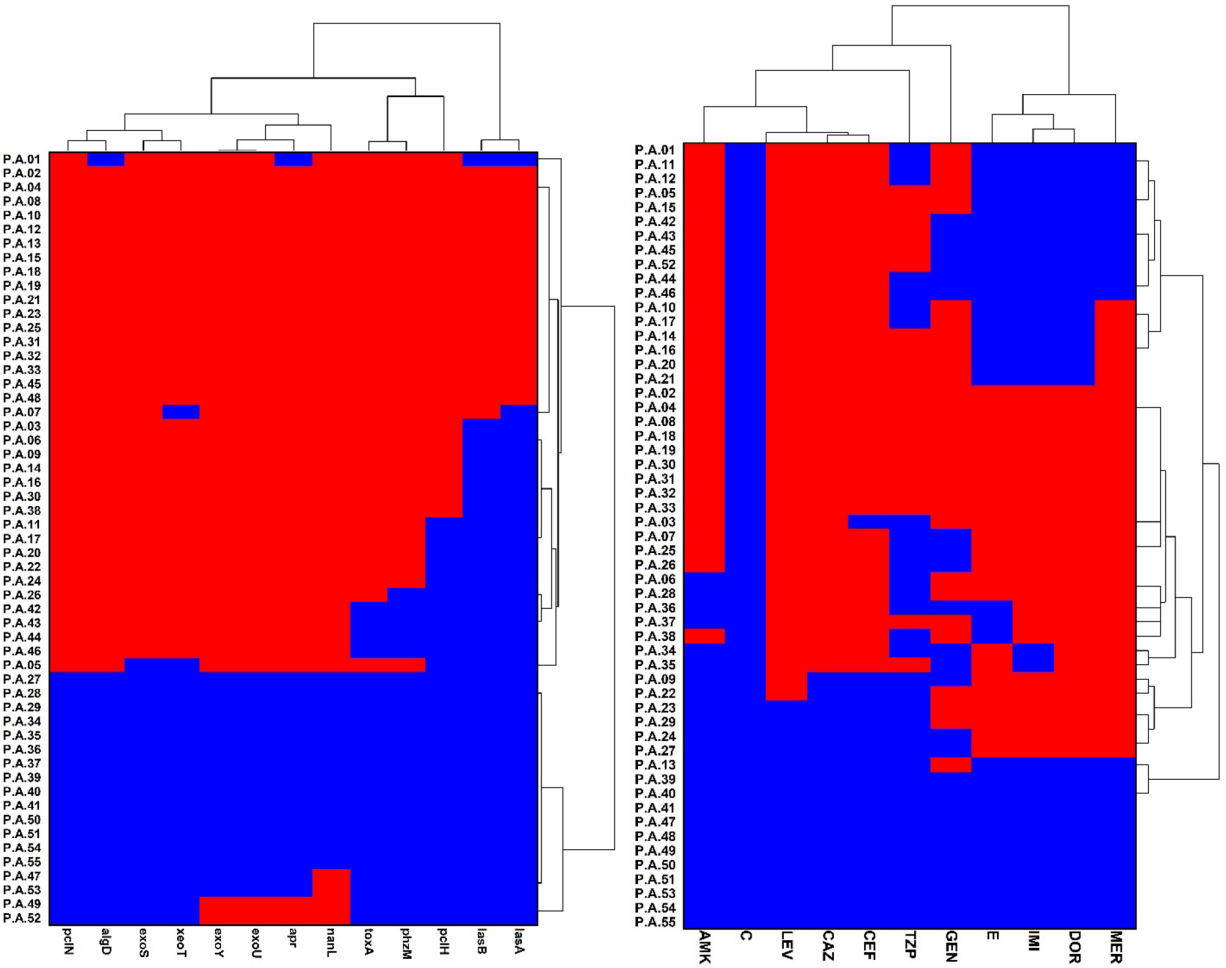
Individual isolates were showing hierarchical clustering of isolates and factors. Binary factors such as antibiotics resistance pattern (right side) and virulence genes (left side) indicating presence as red and absence as green. Clustering is based on the Wald-like test (D_2_) and for binary data.

Antibiotic resistance patterns were presented in Fig. 1. Out of 55 isolates of *P. aeruginosa*, 52 (94.5%) isolates were sensitive to colistin. However, 23 isolates (41.8%) were resistant to doripenem and ceftazidime. Thirty-seven (67.2%) isolates were resistant to at least seven antimicrobials and were considered MDR strains. Further, 18 (32.7%) and nine isolates (16.3%) were considered as XDR and PDR strains, respectively.

### The frequency of biofilm-forming isolates

**Figure 2:**
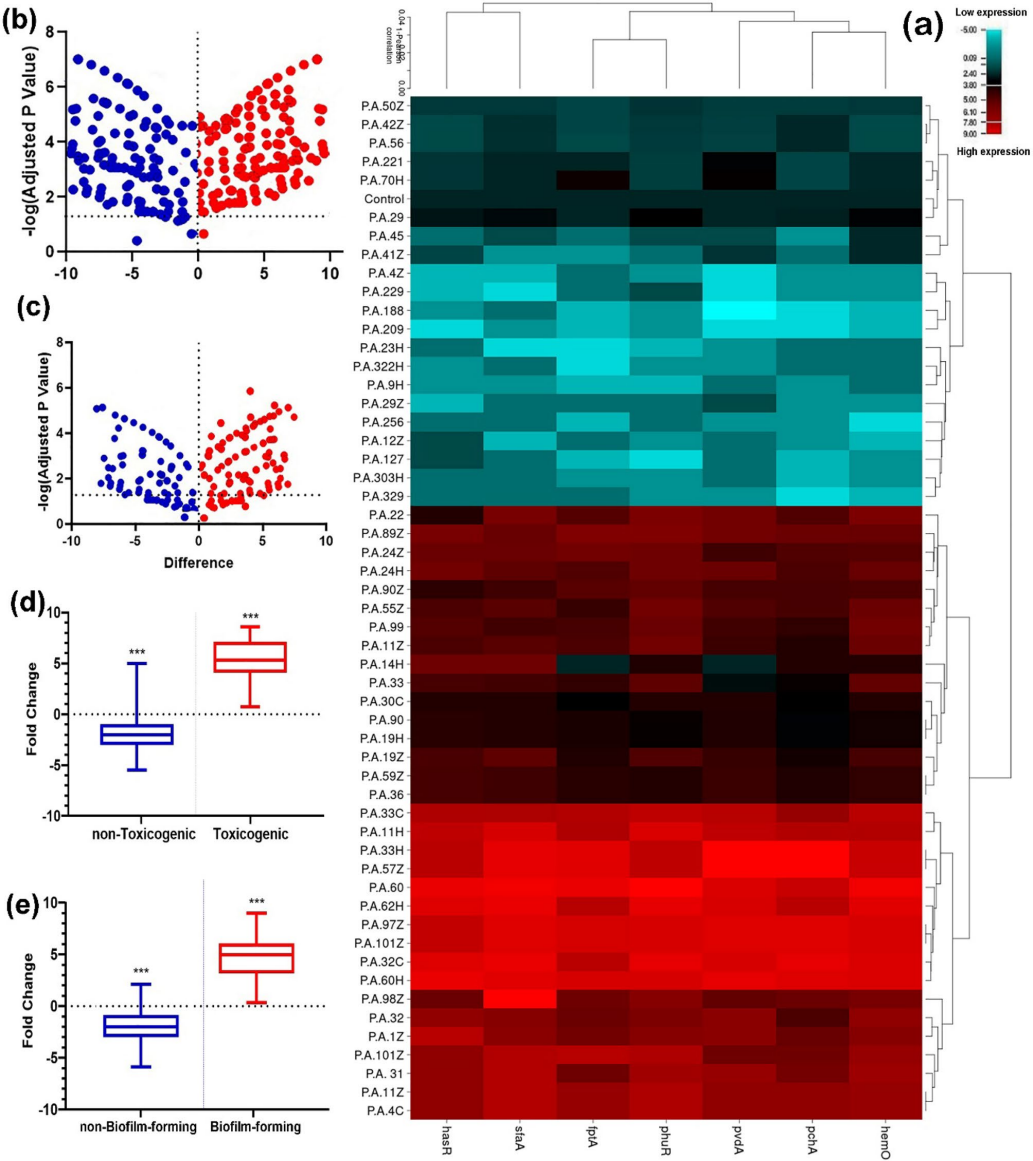
Differential expression of iron-uptake and siderophore system in wound infection isolates of *P. aeruginosa.* Isolates with the potential to form biofilm, non-biofilm-forming, versus toxigenic and non-toxigenic strains. (**a**) Heatmap iron-uptake and siderophore system expression patterns in all 55 *P. aeruginosa*. Blue represents up-regulation and red down-regulation relative to control. (**b**) The volcano plots of iron-uptake and siderophore system genes expressions between toxigenic and non-toxigenic isolates. Expressions analysis based on p-value and log_2_ (fold-change) at an α level of 0.05 and. (**c**) The volcano plots of iron-uptake and siderophore system genes expressions between biofilm and non-biofilm forming isolates, discriminated based on p-value and log_2_ (fold-change) at an α level of 0.05. (**d**) Expression levels of iron-uptake and siderophore system genes between toxigenic a non-toxigenic isolate. Expressions analysis based on p-value and log_2_ (fold-change) at an α level of 0.05. (**e**): Expression levels of iron-uptake and siderophore system genes between biofilm non-biofilm forming isolates, discriminated based on p-value and log_2_ (fold-change) at an α level of 0.05. Error bars standard errors: 0.05. Student’s t-test and Tow-Way ANOVA test were performed for testing differences between groups. *p < 0.05, **p < 0.001, ***p < 0.0001.

The prevalence of biofilm-forming *P. aeruginosa* is shown in Table 1 and Fig. 2. Out of the 55 isolates, 28 (50.9%) isolates were found to form strong biofilms, ten isolates (18.1%) moderately formed biofilm, and 19 isolates (34.5%) were biofilm non-producers.

**Table 1:** Frequency of antibiotic resistance, and virulence factors in biofilm producing and non-biofilm producing *P. aeruginosa* wound collection.

Antimicrobial agent	Biofilm forming isolates (*n* = 37)						Non- biofilm forming isolates (*n* = 18)						*P*^*#*^
	Gender						Gender						
	Female (*n* = 22)			Male (*n* = 15)			Female (*n* = 12)			Male (*n* = 6)			
	R	I	S	R	I	S	R	I	S	R	I	S	
Amikacin	22	0	0	9	4	2	0	0	12	0	0	6	0.064
Gentamycin	22	0	0	7	1	7	0	0	12	0	0	6	0.013
Erythromycin	20	9	4	4	3	8	0	0	12	0	0	6	0.033
Imipenem	13	7	2	8	2	5	1	0	12	0	0	6	0.012
Meropenem	22	0	0	7	1	7	1	0	12	0	0	6	0.011
Doripenem	20	0	2	3	6	6	0	0	12	0	0	6	0.022
Colistin	0	3	19	0	0	15	0	0	12	0	0	6	0.65
Ceftazidime	22	0	0	15	0	0	0	0	12	0	0	6	0.040
Piperacillin/tazobactam	19	0	3	3	0	12	0	0	12	0	0	6	0.82
Cefepime	22	0	0	14	1	0	0	0	12	0	0	6	0.010
Levofloxacin	22	0	0	15	0	0	0	0	12	2	0	4	0.025
MDR		19			11			4			0		0.051
XDR		12			5			0			0		0.072
PDR		6			3			0			0		0.39
**Biofilm formation**													
Strong		18			10			0			0		0.036
Moderate		4			6			0			0		0.023
Week and non-production		0			0			10			9		0.050
**Virulence factors**													
*plcN*		21			16			0			0		0.021
*txoY*		20			15			1			3		0.001
*toxA*		22			11			0			0		0.071
*txoS*		20			10			2			4		0.035
*phzM*		22			10			0			0		0.040
*plcH*		20			6			2			0		0.055
*lasB*		17			3			0			0		0.037
*aprA*		22			14			2			0		0.011
*lasA*		11			8			1			0		0.018
*nanI*		22			16			3			1		0.068
*algD*		22			14			0			0		0.018
*txoU*		20			13			5			1		0.049
*txoT*		19			16			0			0		0.014

### The frequency of VF genes

Out of 55 isolates of *P. aeruginosa*, 9 (15.7%) isolates carry all VFs genes. Moreover, the frequency of VF genes was as follows: *nanI* gene in 41 isolates (74.5%), *exoY* gene in 39 isolates (68.4%), *plcN* gene in 37 isolates (64.9%), and *exoS* gene in 36 isolates (63.1%). Total data of the frequency are shown in Fig. 1.

### Molecular analysis of siderophores, heme, and iron regulatory genes

The results of the expression levels of iron-uptake and siderophore regulatory genes are shown in Fig. 2. Overexpression of iron/siderophore regulatory genes in MDR and XDR strains was observed. Moreover, iron-uptake regulatory genes are less than the expression levels in *P. aeruginosa* ATCC27853. Moreover, *hemO*, *hasR,* and *pvdA* genes are highly expressed in the biofilm-forming isolates. In isolate 60H, iron-uptake and siderophore regulatory genes showed higher expression levels. Whereas, in PA49 and PA221, iron-uptake regulatory genes showed down-expression compared to PA31 and PA60H isolates.

### Analysis of hit map tree of gene expression

Figure 2 shows a comparison among the expression levels for the iron-uptake and siderophore regulatory genes in all 55 isolates of *P. aeruginosa*. The *pchA*, *pvdA,* and *fptA* genes had different activities among the clinical isolates. However, these genes showed a slight expression level in some biofilm-forming isolates and a high expression level in some antibiotic-sensitive strains. Siderophore regulatory genes are expressed to a great extent in strong biofilm-forming isolates. These genes were also highly expressed in colistin-intermediate strains. The majority of the iron-uptake regulatory genes were expressed at deficient levels in the non-toxigenic strains of *P. aeruginosa*. Also, siderophore regulatory genes overexpressed in biofilm-forming and toxigenic isolates. The heat map tree shows a significant relationship between antibiotic resistance and the activity of iron-uptake regulatory genes.

### Analysis of MLST dendrogram phylogenic tree

**Figure 3:**
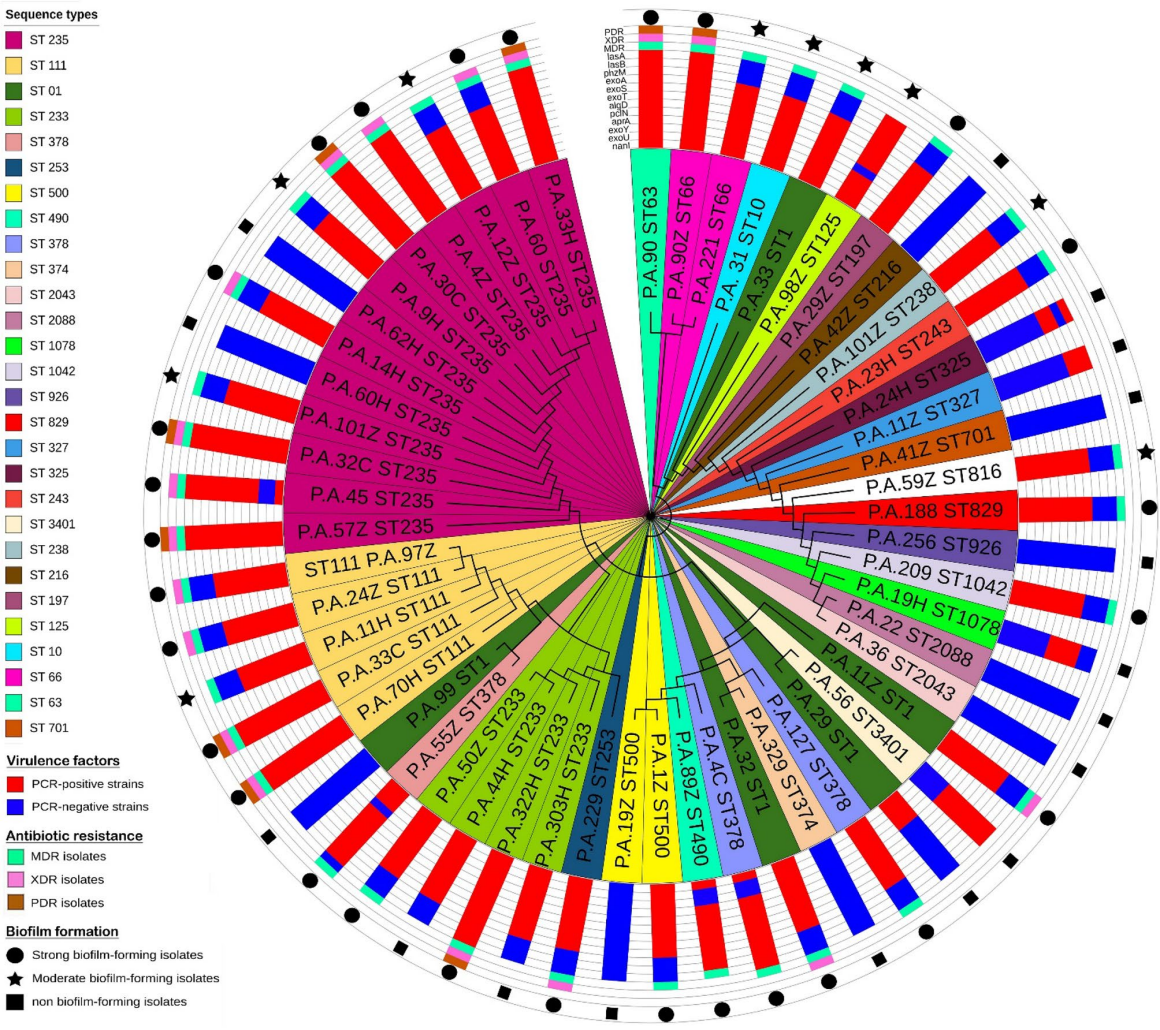
Neighbor-joining, unrooted, circular-dendrogram clustering of 55 isolates of *P. aeruginosa* isolates based on sequence type (ST) profiles. The STs in the same clonal complex are shaded in the same color. The circular-dendrogram was estimated by neighbor-joining using the k2 + G model, with MEGA 6, the tree-drawing tools PHYLIP and the iTOL online tool. Support values are calculated from 500 bootstrap replicates. The STs are shown in a different color.

As shown in Fig. 3, the neighbor-joining tree based on nucleotide difference in sequence data of each housekeeping gene was constructed. Sequencing of housekeeping genes of all 55 representative isolates of *P. aeruginosa* showed 28 sequence types and 28 clusters among all isolates tested. However, ST1, ST235, and ST111 were the most common STs among *P. aeruginosa* isolates. These three STs also showed the highest frequency among MDR and XDR strains. The NBJ tree of the ST235 gene included eight isolates (PA32, PA60H, 97Z, 101Z, 33H, 57Z, 33C, 14H, and 11H) into one significant cluster.

Nineteen unique alleles were identified for each housekeeping gene: 19 for the *guaA* gene*,* 54 for the *nuoD* gene, 39 for *aroE* gene, 33 for the *trpE* gene, 41 for the *ppsA* gene, and 50 for the *guaA* gene. The GC % observed in seven housekeeping genes ranges from 35 to 51%. The sequence types found were ST1, ST10, ST63, ST60, ST125, ST197, ST216, ST29, ST147, ST511, ST70, ST101, ST486, and ST509. ST15, ST111, ST29, ST 147, ST70, and ST101 were among the novel STs from the west of Iran.

### Relationship between virulence factors and iron uptake

**Figure 4:**
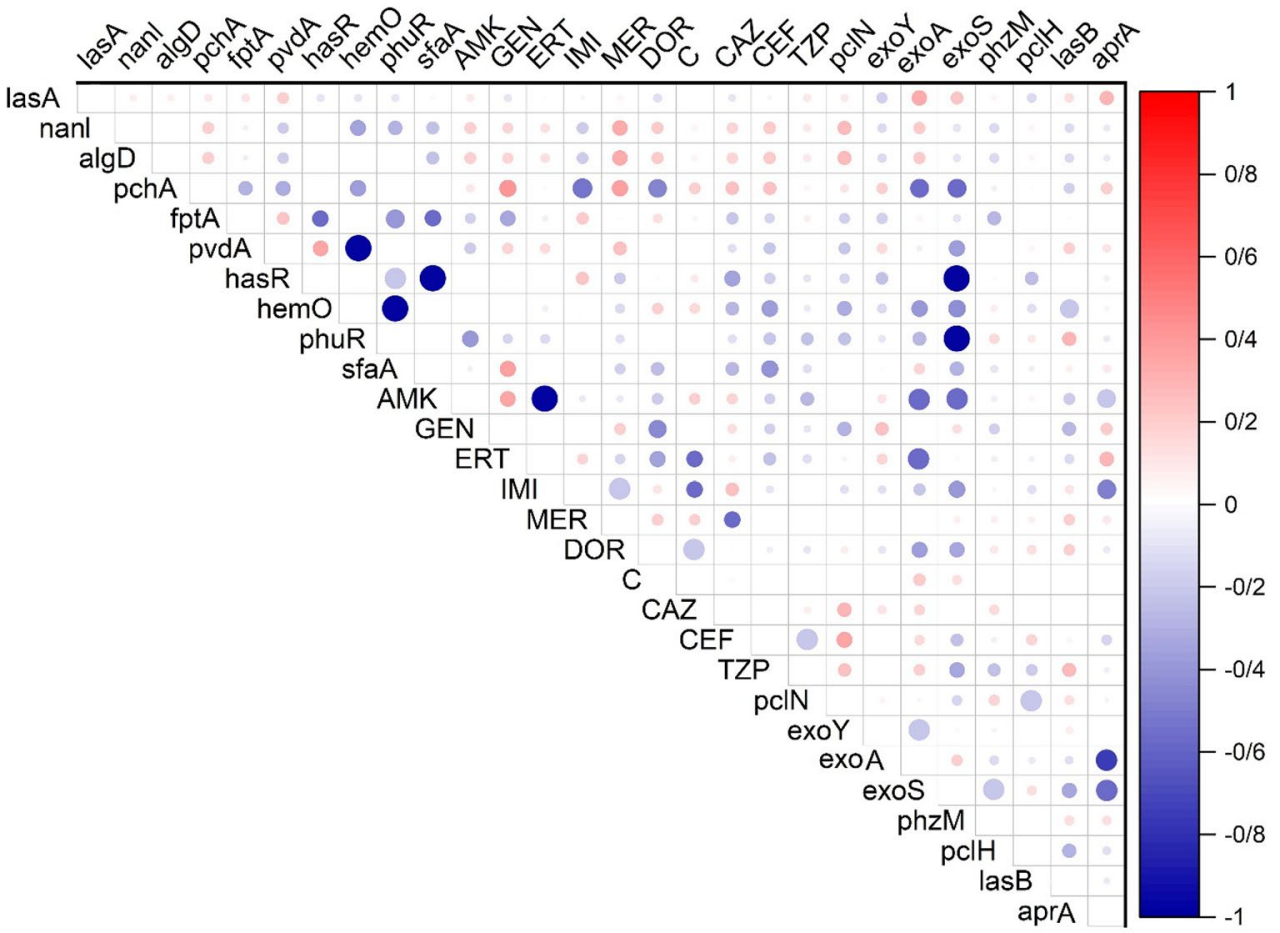
Correlation matrix of phenotypical (antibiotic resistance profile) and genotypical (virulence factor profile and iron/siderophore activity) characteristics with significant correlations (p < 0.05). There is no significant correlation between white spaces. The blue circles indicated a significant positive relationship, and the red indicated a significant negative relationship. The size and strength of color represent the numerical value of the Phi correlation coefficient.

The results of the statical analysis are shown in Table 1 and Fig. 4. The virulence profiles are significantly associated with biofilm formation (p ≤ 0.05). No association between the VFs and colistin, amikacin, and piperacillin/tazobactam resistance was detected. A strong correlation was observed among virulence profiles and expressions of siderophores, heme, and iron regulatory genes (p ≤ 0.001).

## Discussion

In the current study, 94.5% of *P. aeruginosa* were sensitive to colistin, and 67.2% of isolates were resistant to ceftazidime. The observations also agree with the results reported by Nasser et al.^19^. However, some researchers from Nigeria^20^ reported a high frequency of colistin-resistant *P. aeruginosa* strains. The findings of the current study do not support the previous research. However, the mechanisms through which these Gram-negative bacteria acquire polymyxin resistance remain poorly known. Based on these comparisons, the recent appearance of polymyxin resistance highlights the critical need of gaining a better knowledge of the link between various resistance mechanisms and virulence in these Gram-negative bacteria^20^.

In the current study, 66.6% of isolates were able to form a biofilm. These results reflect those of Asati et al. and Kamali et al., who also found that biofilm formation occurs significantly more in burn infections^11,14^.

In the present study, VF profiles of *P. aeruginosa* were detected as *plcN* (67.2%), *exoY* (70.9%), *toxA* (60.0%), *phzM* (58.1%), *pclH* (50.9%), *lasB* (36.3%), *aprA* (69.1%), *lasA* (34.5%), *nanI*(74.5%), *exoU* (70.9%), *exoS* (60.0%), *exoT* (63.6%) and *algD* (65.4%). Our findings agree with Hassuna et al. and Muhsin et al. They found that *exoU*, *exoS* were the most prevalent VFs gene in *P. aeruginosa* isolates^3,21^. These findings further support the idea that VFs play an important role in wound infection caused by *P. aeruginosa*. These results match those observed in earlier studies by Newman et al.^22^.

Based on biofilm results, the expression levels of siderophores, heme, and ferric citrate iron genes on strong biofilm-producing strains were higher than in moderate biofilm producers. There was also a strong correlation between biofilm formation and high expression of siderophores and heme genes. Zhang et al. and Abbas et al. stated that the overexpression of the *tonB* gene in the biofilm-forming strains compared to the non-biofilm-forming strains^7,8^. Poole et al., found that the *tonB* gene plays an essential role in siderophore-mediated iron uptake^23^. In line with the mentioned studies, the fold change of the *tonB* gene in isolates without biofilm (PA49, PA50Z, PA42Z, PA56, PA221, and PA70H) showed the lowest level.

Our results showed the down-regulation of siderophores and heme genes in non-biofilm-forming isolates (PA42Z, PA49, PA221, and PA70H). However, many genes are involved in siderophores, heme, and ferric citrate iron absorption in *P. aeruginosa*. Thus, the bacterium is trying to form a biofilm to preserve its survival against unfavorable factors, using excessive scavenging of iron^19^. The same observations were reported in Ammons and Copié and Kamiya et al., that lactoferrin's high production prevents biofilm formation. However, iron absorption systems help bacteria to form biofilms^24,25^. Moreover, Singh studied the effects of iron on biofilm formation and found that, in the absence of lactoferrin, if no functional iron uptake system were present, biofilms would still form flat, thin colonies^26^.

In the present study, we found that more than 50% of *P. aeruginosa* isolates carried one or more VF genes. Also, VF genes were more abundant in biofilm-forming strains compared to non- biofilm-forming strains. Further, some isolates carrying all or many VF genes (PA11, PA11H, PA101Z, PA60H, PA33C, PA32, and PA31) showed high expression levels of *tonB*, *phuR*, *hemO*, *hasR,* and *pvdA* genes. Some researchers from the USA^27^, Cyprus^15^, and China^28^ also confirmed the association between biofilm formation and increased iron/siderophore regulatory genes. They also showed that *P. aeruginosa* requires iron to form cofactors of enzymes that play essential roles in electron transfer and other critical cellular processes. Iron also signals biofilm formation, but the level of iron necessary to promote biofilm formation exceeds that required for assimilatory purposes. Besides, the production of several virulence determinants, such as exotoxin A, is regulated in response to iron^29^.

Based on the present study, *tonB*, *pchA,* and *pvdA* had a variable expression in toxigenic and non-toxigenic isolates. Also, comparing the value for *pchA* and *fptA* in susceptible isolates to antibiotics with the corresponding value in the resistant isolates, it was observed that the value in the susceptible isolates was consistently lower than the resistant isolates. In non-toxigenic and antibiotic-sensitive strains, biofilm formation may play a direct role in iron uptake and the activity of siderophore controller genes. However, in line with the ideas of Klebba et al., it can be concluded that the *tonB* complex consists of the inner membrane protein *tonB*, which spans the periplasmic space and associates with outer membrane iron receptors^30^. Nonetheless, some researchers from Canada^17^, Germany^31,32^, and Switzerland^18^ proved that *P. aeruginosa* also contains two heme uptake systems whose outer membrane receptors are *HasR* and *PhuR* energized PhuUV ABC transporter *tonB* complex, respectively.

MLST is considered the "gold standard" of typing for many bacterium species. Based on our MLST results, the data generated by nucleotide sequence analysis are unambiguous and easily transferable, and comparable between laboratories, and the methodologies used are both generic and highly reproducible. This method was developed as a comprehensive typing approach that addressed repeatability, reliability, affordability, and throughput requirements^28^. A significant benefit of MLST analysis is that it generates clear sequencing data that are suited for population structure and epidemiological investigation^26,27^.

According to our observation in wound infections, dangerous STs like ST11, ST1, and ST235 had high expression levels of siderophores, heme, and ferric citrate iron genes. These STs have been frequently reported in wound infections, and high levels of antibiotic resistance have also been seen in this ST. In a study, Kim et al.^33^ found that wound infection isolates of *P. aeruginosa* were highly pathogenic and extremely damaged skin tissue. They also showed the overexpression of siderophores and heme genes in isolates of wound infections. ST11 and ST235 were identified as dangerous strains in wound infections in studies by Koutsogiannou et al.^34^ and Omar et al.^35^.

In this study, among 55 isolates, 28 sequence types were detected, and ST1, ST235, and ST111 were the most common STs in *P. aeruginosa* isolates. Moreover, the remaining 12 isolates were found to belong to ST235. These data are in good accordance with what Guzvinec et al.^36^ reported for a collection of *P. aeruginosa* isolates, showing that ST11 and ST253 were more common STs in *P. aeruginosa* isolates. Although most of the ST253 and ST1 in this study were among the most founded STs worldwide, none of these isolates have the same VF profile, and most of them are considered a toxigenic strain. Moreover, ST3401, ST2088, ST2088, ST1078, and ST926 considered non-toxigenic strains. Our current findings are consistent with other studies from Iran^37^, Brazil^38^, and France^39^, where they found ST111, ST235, and ST233 as predominant STs from biofilm-forming *P. aeruginosa* isolates.

However, ST1, ST235, and ST11 also showed the highest frequency among the MDR and XDR strains. The NBJ tree of the ST235 gene included eight isolates (PA32, PA32, PA60H, 97Z, 11H, 101Z, 33H, 57Z, 33C, 14H, 11Z, and 11H) into one central cluster. Interestingly, some sequence types were among the novel STs from the west of Iran. Similar to our results, some STs and variants were seen among resistant and virulent isolates elsewhere by Annear et al.^40^ and Liu et al.^41^; they also identified ST1, ST235 mostly found in hyper pathogenic isolates.

Our data analysis indicated a significant correlation between VF genes and the expression of siderophores, heme, and ferric citrate iron genes (p ≤ 0.001). However, the VF gene profiles are significantly associated with biofilm formation (p ≤ 0.05). Similarly, VF genes were more prevalent in biofilm-forming strains. However, there are no accurate reports of the frequency of sequence typing of *P. aeruginosa* isolates in Iran. Like our results, there have been various reports of those associations in Italy^13^ and Belgium^42^.

In conclusion, our knowledge of this study showed a strong association between regulation genes of siderophores, heme, and ferric citrate iron uptake and biofilm formation. Also, the uptake pathway of siderophore/iron in *P. aeruginosa* plays a significant role in bacterial pathogenesis. Therefore, in chronic wound infections, biofilm formation by siderophores, heme, and ferric citrate iron uptake occurs in *P. aeruginosa*. However, discovering a new clonal complex in a wound infection of *P. aeruginosa* without the previously recognized predisposing factors for the emergence of such strains; has led to a revision of our understanding of wound infection clonal complex epidemiology. Finally, molecular typing can simplify identifying appropriate infection control measures to lower the mortality and morbidity of wound infections.

## Materials and methods

### Ethics statement

The study was approved by the institutional review boards from the Ethics Committee of Hamadan University of Medical (No: IR.UMSHA.REC.1398.481), which allowed the phenotypic and genotypic characterizations of the Hypervirulent *P. aeruginosa* isolates. The project was not involved in the collection and analysis of the demographics and clinical information of any patient. Informed written consent was obtained from the study population or their guardians after providing a full explanation of the study.

### Study design and collection of isolates

Our study was conducted at Hamadan, Iran Hospitals (Farshchian (Sina) Hospital, Be'sat Hospital, and Beheshti Hospital) with about 1000 beds, serving a population in a large metropolitan region. The Hamadan Hospitals and the Hamadan microbiology department have a high reputation in town, ranking first in various medical indicators.

The study included patients with complicated wound infection symptoms (chronic or acute wounds), in Burn Departments of our center who had received carbapenems and other antibiotics before admission or during their hospital stay. Inclusion criteria were treated with antibiotics for at least 24 h and the presence of positive microbiological cultures. Exclusion criteria were negative microbiological cultures or no culture request and multiple or unknown origins of the infection. In Microsoft Office Excel 2019 (Microsoft Corporation, Redmond, WA, USA), stratified sampling was utilized to extract isolates from each layer, and random sampling was employed to acquire a total of burn wound sample isolates.

In the present study, 55 *P. aeruginosa* were isolated from different burn wound samples (384 samples) between July 2019 and April 2020 at Hamadan Hospitals. Burn wound swabs were collected in aseptic conditions, and samples were transported to the microbiology department. Swabs were immediately inoculated on MacConkey Agar (Hi-Media, India). Organisms were identified using Cetrimide Agar (Hi-Media, India) and standard biochemical tests. Finally, all isolates were stored at − 80 °C in Luria–Bertani (Sigma Aldrich) containing 10% glycerol (W/V) (Sigma Aldrich). All methods which used in this study were carried out following relevant guidelines and regulations which were approved by the microbiology department, Hamadan university of medical sciences.

### Antimicrobial susceptibility testing (AST)

The AST was performed using the disk diffusion method (DDM) based on the Clinical & Laboratory Standards Institute (CLSI) guidelines version 2020 for all *P. aeruginosa* isolates^38^. The Liofilchem® MIC Test Strips (Liofilchem, Italy) was used to determine the minimum inhibitory concentration to identify colistin-resistant strains. *P. aeruginosa* ATCC 15442 and *P. aeruginosa* ATCC 27853 were used as control strains^43^.

### Phenotypic screening of biofilm-forming strains

The crystal violet method (CVM) for the screening of biofilm-forming strains was done according to the Manandhar et al. study^44^. The optical density (O.D.) of each well was measured at 570 nm using an ELISA reader (BioTek Instruments, Inc, USA). As the bacteria form biofilm and adhere to the wells, these OD values were taken as bacterial adherence index. The standard strain *Staphylococcus epidermidis* ATCC 35984 was used as a control for biofilm production. The standard strain *Staphylococcus epidermidis* ATCC 12228 was used as the non-biofilm producer control strain.

### DNA extraction and detection of VF genes

The boiling method was used for DNA extraction according to Dehbashi et al. study^2^. Nanodrop (Hangzhou Allsheng Instruments Co., Ltd, China) was used to measure DNA concentration. For screening, the VF genes were performed using the specific primers listed in Table 2 were used. The programmable thermal cycler (Eppendorf, Germany) PCR device was applied in all PCR reactions. The 25 μl reaction mixture contained 12.5 µl of master mix (Ready Mix TM-Taq PCR Reaction Mix, Ampliqon, Denmark), 0.5 μM concentration of each primer, one μl of the 5 ng/μl genomic DNA template, and 11.5 µL of molecular biology grade water. In each round of amplification, sterile water was used as a negative control.

**Table 2:** Primers used for identification of virulence factors and siderophores, heme, and ferric citrate iron uptake genes in wound collection of *P. aeruginosa.*

Genes	Primers	Product Size (bp)	Refs.
*plcN*	F: TCCGTTATCGCAACCAGCCCTACG R: TCGCTGTCGAGCAGGTCGAAC	481	^17^
*exoY*	F: TATCGACGGTCATCGTCAGGT R: TTGATGCACTCGACCAGCAAG	1035	^17^
*exoA*	F: GATGCTGGACGGGTCGAG R: GCACGTGGTCATCCTGATGC	270	^17^
*exoS*	F: CTTGAAGGGACTCGACAAGG R: TTCAGGTCCGCGTAGTGAAT	504	^17^
*phzM*	F: ATGGAGAGCGGGATCGACAG R: ATGCGGGTTTCCATCGGCAG	875	^17^
*plcH*	F: TCCGTAGGCGTCGACGTAC R: TCCGTTATCGCAACCAGCCCTACG	608	^17^
*lasB*	F: GGAATGAACGAGGCGTTCTC R: GGTCCAGTAGTAGCGGTTGG	300	^17^
*aprA*	F: TGTCCAGCAATTCTCTTGC R: CGTTTTCCACGGTGACC	1017	^17^
*lasA*	F: GCAGCACAAAAGATCCC R: GAAATGCAGGTGCGGTC	1075	^17^
*nanI*	F: ATGAATACTTATTTTGATAT R: CTAAATCCATGCTCTGACCC	1317	^17^
*algD*	F: ATGCGAATCAGCATCTTTGGT R: CTACCAGCAGATGCCCTCGGC	1310	^17^
*exoU*	F: GATTCCATCACAGGCTCG R: CTAGCAATGGCACTAATCG	3.038	^17^
*exoT*	F: CAATCATCTCAGCAGAACCC R: TGTCGTAGAGGATCTCCTG	1159	^15^
*pchA*	F: CTGCCTGTACTGGGAACAGC R: GCAGAGCAATTGCCAGTTTT	118	^2^
*fptA*	F: GACTACAGCGTCGACTACCG R: GACCACGCGCCAGCAACCCG	420	^7^
*pvdA*	F: TGTTCCACCACAGCCAGTAC R: GGGTAGCTGTCGTTGAGGTC	133	^24^
*hasR*	F: CTGGCGTCGAGTACCAG R: GGTCTTCGAACAGAAGTCGTTG	99	^23^
*hemO*	F: TGGTGAAGAGCAAGGAACCCTTC R: TTCGTTGCGATAAAGCGGCTCCA	104	^23^
*phuR*	F: ACTGCCCAACGACTTCTTCAG R: TTACGATGTCCGGATCGACGTA	71	^23^
*tonB*	F: CCTGCCATGCGTGAATGC R: AGAACATCTTGGTCGCCTGG	203	^25^

S, susceptible; R, resistant; I, intermediate.

^#^Statistical relationship between chi-square test between different variables with significant level ≤ 0.05.

### RNA extraction, synthesis of cDNA, and quantitative real-time PCR (qRT-PCR)

Total RNA extraction and synthesis of cDNA were performed according to Dehbashi et al. study^37^. PCR reactions were performed in 96-well microplates (ABI-Step One-Plus) using the ABI-Step One-Plus Real-time System, ABI, USA. qRT-PCR was carried out using 4 μl of 2 × FIREPol Master Mix (Solis BioDyne, Tartu, Estonia), 0.5 μl (10 pM) forward and reverse primers were 2 μl template cDNA, and 13 μl RNase free water to a final volume of 20 μl. The PCR protocol was designed for 40 cycles, and a melting-curve analysis (65–95 °C, fluorescence read every 0.3 °C) was performed to check the amplification specificity. For differential gene expression study, relative quantification was achieved using the CT comparative method (Fold of Expression = 2^−ΔΔCT^)^45^.

### Generating heat maps of expression data

According to the Dehbashi et al. study^46^, the heatmap2 function from the One Matrix CIM online package (https://discover.nci.nih.gov/cimminer/home.do) was used to build heat maps. The hierarchically clustered distance matrix was used as an input for the heatmap function (https://discover.nci.nih.gov/cimminer/home.do).

### MLST of isolates

MLST for *P. aeruginosa* was performed, according to Tahmasebi et al.^47^. MLST of *P. aeruginosa* was performed based on the sequences of seven housekeeping genes *acsA*, *aroE*, *guaA*, *mutL*, *nuoD*, *ppsA*, and *trpE* (https://pubmlst.org/paeruginosa/info/primers.shtml). Allele numbers were determined by comparison with those allele sequences deposited in GenBank. Sequence types (STs) were determined using the key table kindly provided by the *P. aeruginosa* MLST database (https://pubmlst.org/bigsdb?db=pubmlst_paeruginosa_seqdef).

### Statistical analysis

All statistical analyses were performed using GraphPad Prism, version 8.0 (GraphPad Software, Inc., CA, USA). The relationship between categorical variables was compared using the chi-square test or Fisher's exact test. The Student’s t-test was used for continuous variables. Two-way ANOVA was performed for the comparison of normally distributed data. P-values of less than 0.05 were considered statistically significant for all the statistical tests performed. Expression analysis data were taken in three replication and given as mean value ± SE. As recommended by the manufacturer, all baseline and threshold values were reviewed and manually adjusted as required. Further analysis was performed in Data-Assist (Applied Biosystems, CA, USA). The p-value was calculated based on a two-sample, two-tailed Student’s t-test for the calculated Fold change (relative to the epilepsy group).

A dendrogram was constructed from a distance-based matrix of the allelic profiles using the neighbor-joining method. In this study MEGA version 6, the tree-drawing tools PHYLIP (available at http: //pubmlst.org/analysis) and the iTOL online tool (http://itol.embl .de/itol.cgi) were used.

The phenotypic antibiotic susceptibility profiles and the presence of genes were converted to binary code. For each antibiotic, 0 indicated susceptibility, and one (1) indicated resistance; likewise, a gene's presence was designated as one (1) and absence as 0. *P. aeruginosa* from various clinical samples have been carried out using a Heatmap packaging in statistical program R. Principal component analysis was performed using the package Facto-extra from the open-source statistical program R.

### Ethical approval

The ethics committee approved this study of Hamadan University of Medical Sciences (No: IR.UMSHA.REC.1398.481).

## References

[1] Zahedani SS, Tahmasebi H, Jahantigh M (2021). Coexistence of virulence factors and efflux pump genes in clinical isolates of *Pseudomonas aeruginosa*: analysis of biofilm-forming strains from Iran. Int. J. Microbiol..

[2] Dehbashi S, Tahmasebi H (2018). Arabestani MR Association between beta-lactam antibiotic resistance and virulence factors in AmpC producing clinical strains of *P. aeruginosa*. Osong. Public Health Res. Perspect..

[3] Hassuna NA, Mandour SA, Mohamed ES (2020). Virulence constitution of multi-drug-resistant *Pseudomonas aeruginosa* in upper Egypt. Infect Drug Resist..

[4] Mirzaei B, Bazgir ZN, Goli HR, Iranpour F, Mohammadi F, Babaei R (2020). Prevalence of multi-drug resistant (MDR) and extensively drug-resistant (XDR) phenotypes of *Pseudomonas aeruginosa* and *Acinetobacter baumannii* isolated in clinical samples from Northeast of Iran. BMC Res. Notes..

[5] Meyer JM, Abdallah MA (1978). The fluorescent pigment of pseudomonas fluorescens: Biosynthesis purification and physicochemical properties. Microbiology..

[6] Leoni L, Ciervo A, Orsi N, Visca P (1996). Iron-regulated transcription of the pvdA gene in *Pseudomonas aeruginosa*: Effect of Fur and PvdS on promoter activity. J. Bacteriol..

[7] Abbas A, Adams C, Scully N, Glennon J, O'Gara F (2007). A role for TonB1 in biofilm formation and quorum sensing in *Pseudomonas aeruginosa*. FEMS Microbiol. Lett..

[8] Zhang Y, Gao J, Wang L, Liu S, Bai Z, Zhuang X (2018). Environmental adaptability and quorum sensing: Iron uptake regulation during biofilm formation by *Paracoccus denitrificans*. Appl. Environ. Microbiol..

[9] Schalk IJ, Guillon L (2013). Pyoverdine biosynthesis and secretion in *Pseudomonas aeruginosa*: Implications for metal homeostasis. Environ. Microbiol..

[10] Visca P, Imperi F, Lamont IL (2007). *Pyoverdine siderophores*: From biogenesis to biosignificance. Trends Microbiol..

[11] Asati S, Chaudhary U (2017). Prevalence of biofilm producing aerobic bacterial isolates in burn wound infections at a tertiary care hospital in northern India. Ann. Burns Fire Disasters.

[12] Ankenbauer RG, Quan HN (1994). FptA, the Fe(III)-pyochelin receptor of *Pseudomonas aeruginosa*: A phenolate siderophore receptor homologous to hydroxamate siderophore receptors. J. Bacteriol..

[13] Minandri F, Imperi F, Frangipani E, Bonchi C, Visaggio D, Facchini M (2016). Role of iron uptake systems in *Pseudomonas aeruginosa* virulence and airway infection. Infect. Immun..

[14] Kamali E, Jamali A, Ardebili A, Ezadi F, Mohebbi A (2020). Evaluation of antimicrobial resistance, biofilm forming potential, and the presence of biofilm-related genes among clinical isolates of *Pseudomonas aeruginosa*. BMC Res. Notes..

[15] Panayidou S, Georgiades K, Christofi T, Tamana S, Promponas VJ, Apidianakis Y (2020). *Pseudomonas aeruginosa* core metabolism exerts a widespread growth-independent control on virulence. Sci. Rep..

[16] Leoni L, Orsi N, de Lorenzo V, Visca P (2000). Functional analysis of PvdS, an iron starvation sigma factor of *Pseudomonas aeruginosa*. J. Bacteriol..

[17] Poole K, Zhao Q, Neshat S, Heinrichs DE, Dean CR (1996). The *Pseudomonas aeruginosa* tonB gene encodes a novel TonB protein. Microbiology.

[18] Luscher A, Moynié L, Auguste PS, Bumann D, Mazza L, Pletzer D (2018). TonB-dependent receptor repertoire of *Pseudomonas aeruginosa* for uptake of siderophore-drug conjugates. Antimicrob. Agents Chemother..

[19] Nasser M, Gayen S, Kharat AS (2020). Prevalence of β-lactamase and antibiotic-resistant *Pseudomonas aeruginosa* in the Arab region. J. Global Antimicrob. Res..

[20] Umar AI, Garba I, Jidda ML, Ganau AM, Fana AS, Umar AI (2019). Multidrug-resistant Pseudomonas aeruginosa isolated from ear and wound swabs in some selected hospital laboratories in Sokoto metropolis, Nigeria. Calabar J. Health Sci..

[21] Ali AM, Al-Kenanei KA, Hussein SN, Bdaiwi QO (2020). Molecular study of some virulence genes of *Pseudomonas aeruginosa* isolated from different infections in hospitals of Baghdad. Rev. Med. Microbiol..

[22] Newman JW, Floyd RV, Fothergill JL (2017). The contribution of *Pseudomonas aeruginosa* virulence factors and host factors in the establishment of urinary tract infections. FEMS Microbiol. Lett..

[23] Poole K, Young L, Neshat S (1990). Enterobactin-mediated iron transport in *Pseudomonas aeruginosa*. J. Bacteriol..

[24] Ammons MC, Copié V (2013). Mini-review—Lactoferrin: A bioinspired, anti-biofilm therapeutic. Biofouling.

[25] Kamiya H, Ehara T, Matsumoto T (2012). Inhibitory effects of lactoferrin on biofilm formation in clinical isolates of *Pseudomonas aeruginosa*. J. Infect. Chemother..

[26] Singh PK (2004). Iron sequestration by human lactoferrin stimulates *P. aeruginosa* surface motility and blocks biofilm formation. Biometals..

[27] Reinhart AA, Oglesby-Sherrouse AG (2016). Regulation of *Pseudomonas aeruginosa* virulence by distinct iron sources. Genes.

[28] Huang H, Shao X, Xie Y, Wang T, Zhang Y, Wang X (2019). An integrated genomic regulatory network of virulence-related transcriptional factors in *Pseudomonas aeruginosa*. Nat. Commun..

[29] Weigert M, Ross-Gillespie A, Leinweber A, Pessi G, Brown SP, Kümmerli R (2016). Manipulating virulence factor availability can have complex consequences for infections. Evol. Appl..

[30] Klebba PE, Newton SMC, Six DA, Kumar A, Yang T, Nairn BL (2021). Iron acquisition systems of gram-negative bacterial pathogens define TonB-dependent pathways to novel antibiotics. Chem. Rev..

[31] Page MGP (2019). The role of iron and siderophores in infection, and the development of siderophore antibiotics. Clin. Infect. Dis..

[32] Miethke M, Marahiel MA (2007). Siderophore-based iron acquisition and pathogen control. Microbiol. Mol. Biol. Rev..

[33] Kim M, Christley S, Khodarev NN, Fleming I, Huang Y, Chang E (2015). *Pseudomonas aeruginosa* wound infection involves activation of its iron acquisition system in response to fascial contact. J. Trauma Acute Care Surg..

[34] Koutsogiannou M, Drougka E, Liakopoulos A, Jelastopulu E, Petinaki E, Anastassiou ED (2013). Spread of multidrug-resistant *Pseudomonas aeruginosa* clones in a university hospital. J. Clin. Microbiol..

[35] Omar A, Wright JB, Schultz G, Burrell R, Nadworny P (2017). Microbial biofilms and chronic wounds. Microorganisms..

[36] Guzvinec M, Izdebski R, Butic I, Jelic M, Abram M, Koscak I (2014). Sequence types 235, 111, and 132 predominate among multidrug-resistant *Pseudomonas aeruginosa* clinical isolates in Croatia. Antimicrob. Agents Chemother..

[37] Dehbashi S, Tahmasebi H, Alikhani MY, Keramat F, Arabestani MR (2020). Distribution of Class B and Class A β-lactamases in clinical strains of *Pseudomonas aeruginosa*: Comparison of phenotypic methods and high-resolution melting analysis (HRMA) assay. Infect. Drug. Resist..

[38] de Sales RO, Migliorini LB, Puga R, Kocsis B, Severino P (2020). A Core genome multilocus sequence typing scheme for *Pseudomonas aeruginosa*. Front. Microbiol..

[39] Treepong P, Kos VN, Guyeux C, Blanc DS, Bertrand X, Valot B (2018). Global emergence of the widespread *Pseudomonas aeruginosa* ST235 clone. Clin. Microbiol. Infect..

[40] Annear D, Black J, Govender S (2017). Multilocus sequence typing of carbapenem resistant *Pseudomonas aeruginosa* isolates from patients presenting at Port Elizabeth Hospitals, South Africa. Afr. J. Infect. Dis..

[41] Liu H, Kong W, Yang W, Chen G, Liang H, Zhang Y (2018). Multilocus sequence typing and variations in the oprD gene of *Pseudomonas aeruginosa* isolated from a hospital in China. Infect. Drug. Resist..

[42] Cornelis P, Dingemans J (2013). *Pseudomonas aeruginosa* adapts its iron uptake strategies in function of the type of infections. Front. Cell. Infect. Microbiol..

[43] CLSI. 2020. *Performance Standards for Antimicrobial Susceptibility Testing: 30nd Informational Supplement CLSI M100-S30*. CLSI, Wayne, PA.

[44] Manandhar S, Singh A, Varma A, Pandey S, Shrivastava N (2018). Evaluation of methods to detect in vitro biofilm formation by staphylococcal clinical isolates. BMC Res. Notes..

[45] Bustin SA, Benes V, Nolan T, Pfaffl MW (2005). Quantitative real-time RT-PCR–a perspective. J. Mol. Endocrinol..

[46] Dehbashi S, Tahmasebi H, Zeyni B, Arabestani MR (2021). Regulation of virulence and β-lactamase gene expression in *Staphylococcus aureus* isolates: cooperation of two-component systems in bloodstream superbugs. BMC Microbiol..

[47] Tahmasebi H, Dehbashi S, Arabestani MR (2020). Prevalence and molecular typing of colistin-resistant *Pseudomonas aeruginosa* (CRPA) among β-lactamase-producing isolates: A study based on high-resolution melting curve analysis method. Infect. Drug. Resist..

